# Association of carotid and intracranial stenosis with Alzheimer’s disease biomarkers

**DOI:** 10.1186/s13195-020-00675-6

**Published:** 2020-09-10

**Authors:** Koung Mi Kang, Min Soo Byun, Jun Ho Lee, Dahyun Yi, Hye Jeong Choi, Eunjung Lee, Younghwa Lee, Jun-Young Lee, Yu Kyeong Kim, Bo Kyung Sohn, Chul-Ho Sohn, Dong Young Lee, for the KBASE Research Group

**Affiliations:** 1 Department of Radiology, Seoul National University Hospital, 101 Daehak-ro, Jongno-gu, Seoul, 03080 Republic of Korea; 2 Department of Neuropsychiatry, Seoul National University Bundang Hospital, Seongnam, Republic of Korea; 3 Department of Neuropsychiatry, National Center for Mental Health, Seoul, Republic of Korea; 4 Institute of Human Behavioral Medicine, Medical Research Center Seoul National University, Seoul, Republic of Korea; 5 Department of Radiology, CHA Bundang Medical Center, CHA University, Seongnam, Republic of Korea; 6 Department of Radiology, Chung-Ang University Hospital, Seoul, Republic of Korea; 7 Department of Neuropsychiatry, Seoul National University Hospital, 101 Daehak-ro, Jongno-gu, Seoul, 03080 Republic of Korea; 8 Department of Neuropsychiatry, SMG-SNU Boramae Medical Center, Seoul, Republic of Korea; 9 Department of Psychiatry, Seoul National University College of Medicine, 101 Daehak-ro, Jongno-gu, Seoul, 03080 Republic of Korea; 10 Department of Nuclear Medicine, SMG-SNU Boramae Medical Center, Seoul, Republic of Korea; 11 Department of Psychiatry, Sanggye Paik Hospital, Inje University College of Medicine, Seoul, Republic of Korea; 12 Department of Radiology, Seoul National University College of Medicine, 101 Daehak-ro, Jongno-gu, Seoul, 03080 Republic of Korea

**Keywords:** Alzheimer’s disease, Amyloid beta, Neurodegeneration, Atherosclerosis, Intracranial stenosis, Carotid stenosis, Cognitive impairment

## Abstract

**Background:**

To clarify whether atherosclerosis of the carotid and intracranial arteries is related to Alzheimer’s disease (AD) pathology in vivo, we investigated the associations of carotid and intracranial artery stenosis with cerebral beta-amyloid (Aβ) deposition and neurodegeneration in middle- and old-aged individuals. Given different variations of the pathologies between cognitive groups, we focused separately on cognitively normal (CN) and cognitively impaired (CI) groups.

**Methods:**

A total of 281 CN and 199 CI (mild cognitive impairment and AD dementia) subjects underwent comprehensive clinical assessment, [^11^C] Pittsburgh compound B-positron emission tomography, and magnetic resonance (MR) imaging including MR angiography. We evaluated extracranial carotid and intracranial arteries for the overall presence, severity (i.e., number and degree of narrowing), and location of stenosis.

**Results:**

We found no associations between carotid and intracranial artery stenosis and cerebral Aβ burden in either the CN or the CI group. In terms of neurodegeneration, exploratory univariable analyses showed associations between the presence and severity of stenosis and regional neurodegeneration biomarkers (i.e., reduced hippocampal volume [HV] and cortical thickness in the AD-signature regions) in both the CN and CI groups. In confirmatory multivariable analyses controlling for demographic covariates and diagnosis, the association between number of stenotic intracranial arteries ≥ 2 and reduced HV in the CI group remained significant.

**Conclusions:**

Neither carotid nor intracranial artery stenosis appears to be associated with brain Aβ burden, while intracranial artery stenosis is related to amyloid-independent neurodegeneration, particularly hippocampal atrophy.

## Background

Clinical or epidemiological studies indicate the association between atherosclerosis of the carotid and intracranial arteries and Alzheimer’s disease (AD) dementia [1–4]. However, the pathological links underlying the association still remain unclear. Although some postmortem studies reported the relationship between atherosclerosis and amyloid plaques, a core pathology of AD [5–8], others did not find such relationship [4, 9–11]. Regarding in vivo brain imaging approaches, a couple of small-scale studies on individuals with severe cerebral hypoperfusion yielded inconsistent findings on the relationship between very severe atherosclerosis and beta-amyloid (Aβ) deposition [12, 13]. A recent study using high-resolution vessel wall magnetic resonance (MR) imaging reported that intracranial atherosclerotic plaque or stenosis was not associated with Aβ deposition in nondemented adults [14]. Nevertheless, knowledge of the association between carotid and intracranial atherosclerosis and other in vivo brain pathologies including regional neurodegeneration, as well as Aβ deposition, in the living human brain remains limited.

The relationships between carotid and intracranial artery atherosclerosis and in vivo brain pathologies may be different according to the cognitive status of individuals because of the following reasons: regional neurodegeneration is minimal in cognitively normal (CN) individuals, resulting in floor effect, while it has wide variation in those with cognitive impairment (CI), correlating with the degree of cognitive decline [15]. In addition, many AD dementia patients show almost saturated Aβ deposition, possibly resulting in ceiling effect [15]. Given these reasons, a cognitive state-specific approach, separately focusing on CN and CI individuals, could be helpful.

We aimed to investigate the associations between carotid and intracranial artery stenosis systematically measured on MR angiography and AD biomarkers including cerebral Aβ deposition and regional neurodegeneration in a large number of older adults including both the CN and CI groups.

## Methods

### Participants

This study is part of the Korean Brain Aging Study for Early Diagnosis and Prediction of Alzheimer’s Disease (KBASE), an ongoing prospective, community-based cohort study [16]. As of February 2017, a total of 480 older adults consisting of 281 CN and 199 CI (MCI and AD dementia) subjects were initially recruited. The inclusion criteria for the CN group were (a) aged 55–90 years, (b) no diagnosis of MCI or dementia, and (c) Clinical Dementia Rating (CDR) score of 0. For the MCI group, individuals 55–90 years old who fulfilled the core clinical criteria for diagnosis of MCI according to the recommendations of the National Institute on Aging-Alzheimer’s Association (NIA-AA) guidelines [17] were included as follows: (a) memory complaints corroborated by the patient, an informant, or clinician; (b) objective memory impairment for age, education, and gender (i.e., at least 1.0 SD below the respective age, education, and gender-specific mean for at least one of the four episodic memory tests included in the Korean version of Consortium to Establish a Registry for Alzheimer’s Disease (CERAD-K) neuropsychological battery [Word List Memory, Word List Recall, Word List Recognition, and Constructional Recall test]); (c) largely intact functional activities; and (d) no dementia. The global CDR score of all MCI individuals was 0.5. For the AD dementia group, participants 55–90 years old who fulfilled the following inclusion criteria were recruited: (a) criteria for dementia in accordance with the Diagnostic and Statistical Manual of Mental Disorders 4th Edition (DSM-IV-TR), (b) the criteria for probable AD dementia in accordance with the NIA-AA guidelines [18], and (c) a global CDR score of 0·5 or 1. For all groups, individuals with the following conditions were excluded from the study: (1) presence of major psychiatric illness; (2) significant neurological or medical condition or comorbidities that could affect mental function; (3) contraindications to MRI (e.g., pacemaker, claustrophobia); (4) illiteracy; (5) presence of significant visual/hearing difficulty, and severe communication or behavioral problems that would make clinical examination or brain scan difficult; (6) taking an investigational drug; and (7) pregnant or breastfeeding. More detailed information on recruitment of the KBASE cohort was described in our previous report [16]. This study protocol was approved by the Institutional Review Boards of Seoul National University Hospital and SNU-SMG Boramae Medical Center, Seoul, South Korea. The participants and/or their legal representatives provided written informed consent.

### Clinical assessment

All participants were administered comprehensive clinical and neuropsychological assessments by trained psychiatrists and neuropsychologists based on the KBASE assessment protocol which incorporates the CERAD-K [16]. Blood samples were collected to determine apolipoprotein E ε4 allele (APOE4) carrier status. Vascular risk factors, including hypertension, diabetes mellitus, hyperlipidemia, coronary artery disease, transient ischemic attack, and stroke, were evaluated via systematic interview by trained nurses, and vascular risk score was calculated for the number of vascular risk factors present and reported as a percentage [16].

### Image acquisition, preprocessing, and measurement of vessel stenosis and AD biomarkers

All subjects underwent simultaneous three-dimensional (3D) [^11^C] Pittsburgh compound B (PiB)-positron emission tomography (PET), 3D T1-weighted MRI, fluid-attenuated inversion (FLAIR) images, and 3D time-of-flight (TOF)-MR angiography using the 3.0-T Biograph mMR (PET-MR) scanner (Siemens, Washington DC, USA). Acquisition parameters for MRI and MR angiography are described in Methods S1 (Additional file 1).

#### Systematic evaluation of stenosis on MR angiography

Diagnosis of extracranial carotid and intracranial arterial stenosis was reached by the consensus between two qualified neuroradiologists (KMK and CHS) blinded to the participants’ clinical information. We recorded the overall presence, the number, and the degree of detectable stenotic lesions in the following 13 arterial segments: right and left proximal cervical internal carotid artery (ICA); right and left intracranial ICA; right and left anterior, middle, and posterior cerebral arteries; right and left intracranial vertebral artery; and basilar artery. For the extracranial carotid artery, the degree of stenosis was measured according to the North American Symptomatic Carotid Endarterectomy Trial (NASCET) criteria [19] using maximum-intensity projections and source images of the bifurcation of the carotid artery. In cases of intracranial arterial stenosis, the degree of stenosis was calculated based on maximum-intensity projections and source images using the method published for the Warfarin-Aspirin Symptomatic Intracranial Disease Study [20]: percent stenosis = [(1 − (*D*_stenosis_/*D*_normal_)] × 100. In the case of an artery with multiple stenotic lesions, the most severe degree was selected. Based on the above quantitative data, participants were categorized into stenosis-positive (stenosis+) vs. stenosis-negative (stenosis−) groups according to the stenosis measurements for extracranial carotid and intracranial arteries as follows: (i) overall presence of any detectable stenosis and (ii) severity (i.e., the degree of stenosis ≥ 50%, and number of stenotic arteries ≥ 2). In terms of the location of intracranial arterial stenosis, the presence of detectable stenosis in the anterior circulation and posterior circulations was also evaluated. Anterior circulation stenosis was defined as any detectable stenosis in ICA and anterior or middle cerebral arteries. Posterior circulation stenosis included any detectable stenosis in intracranial vertebral or basilar arteries. As there were only very limited numbers of cases with ≥ 50% stenotic lesions in the extracranial carotid arteries (1 of 281 subjects in the CN group and 1 of 196 subjects in the CI group), and those with bilateral extracranial carotid stenosis (6 of 281 subjects in the CN group and 6 of 196 subjects in the CI group) in our sample, these measurements could not be applied to the extracranial carotid arteries and only available for evaluation of intracranial arterial stenosis.

#### Beta-amyloid (Aβ) biomarker

For measurement of Aβ biomarker of AD, a 30-min emission scan was obtained 40 min after injection of intravenous administration of 555 MBq of [^11^C] PiB (range, 450–610 MBq). The [^11^C] PiB-PET data collected in list mode were processed for routine corrections such as uniformity, UTE-based attenuation, and decay corrections, and were reconstructed into a 344 × 344 image matrix using iterative methods (5 iterations with 21 subsets). The image preprocessing steps were performed using Statistical Parametric Mapping 8 (SPM8; http://www.fil.ion.ucl.ac.uk/spm) implemented in MATLAB 2014a (MathWorks, Natick, MA, USA). Static [^11^C] PiB-PET images were coregistered to individual T1 structural images, and transformation parameters for spatial normalization of individual T1 images to a standard Montreal Neurological Institute (MNI) template were calculated. The inverse transformation of parameters to transform coordinates from the automatic anatomic labeling (AAL) 116 atlas [21] into an individual space for each subject (resampling voxel size = 1 × 0.98 × 0.98 mm) was performed using IBASPM (Individual Brain Atlases using Statistical Parametric Mapping) software in MATLAB. To extract gray matter (GM) and exclude the non-GM portions of the atlas (i.e., white matter [WM] and cerebrospinal fluid space), a GM mask, which is a binary probabilistic GM map generated by preprocessing step using SPM8, was applied for each individual. The mean regional [^11^C] PiB uptake values from cerebral regions were extracted using the individual AAL 116 atlas from T1-coregistered [^11^C] PiB-PET images. Cerebellar GM was used as the reference region for quantitative normalization of cerebral [^11^C] PiB uptake values, due to its relatively low Aβ deposition [22], with a probabilistic cerebellar atlas (Institute of Cognitive Neuroscience, UCL; Cognitive Neuroscience Laboratory, Royal Holloway, University of London, UK) which was transformed into individual space as described above. The AAL algorithm and a region combining method [23] were applied to determine regions of interest (ROIs) to characterize the [^11^C] PiB retention levels in the frontal, lateral parietal, posterior cingulate-precuneus, and lateral temporal regions. A global Aβ retention value (standardized uptake value ratio, SUVR) was generated by dividing the voxel-weighted mean value of the four ROIs by the mean cerebellar uptake value [23–25]. Aβ positivity was defined if [^11^C] PiB SUVR value was > 1.4 in at least one of the abovementioned four ROIs [23–25].

#### Neurodegeneration biomarker

All T1-weighted MR images were automatically segmented using FreeSurfer version 5.3 (http://surfer.nmr.mgh.harvard.edu/) with manual correction of minor segmentation errors. As AD-related neurodegeneration biomarkers, both AD-signature cortical thickness (AD-CT; i.e., mean cortical thickness obtained from AD-signature regions) and hippocampal volume adjusted for intracranial volume (HVa) were measured as described previously [24]. First, AD-CT was defined as the mean cortical thickness values of AD-signature regions including the entorhinal, inferior temporal, middle temporal, and fusiform gyrus, based on the Desikan–Killiany atlas [26]. Second, to obtain HVa, left and right hippocampi volume from the FreeSurfer extracted output were first added together to yield the total hippocampal volume (HV). Then, the volume deviating from the expected total HV according to intracranial volume (ICV) based on the reference group (i.e., young CN group of the study cohort [KBASE]), which was not included in the present study, was calculated to obtain HVa as described in the previous study [24, 27]. Briefly, a linear regression line was derived based on the young control group (i.e., the reference group) using their ICV and total HV. HVa, then, is the residual from the linear regression of HV (*y*-axis) vs. ICV (*x*-axis); therefore, the HVa is interpreted as the amount of deviation in a subject’s hippocampal volume from what is expected given their ICV.

#### White matter hyperintensities

The volume of white matter hyperintensities (WMH) on FLAIR images was calculated using a validated automatic procedure [28] with two modifications, as follows: First, an optimal threshold of 70 instead of 65 in the original reference was applied, as the original study [28] recommended adjustment of the threshold for each study dataset to capture the voxels with WMH better than 65 without including non-WMH voxels. More detailed information on the adjustment of the threshold is described in Methods S2 (Additional file 1). Second, diffusion-weighted imaging was not used in the present automated procedure as there were no participants with acute cerebral infarcts in our study population. WMH candidate images were used to extract WMH volumes based on lobar ROIs in the native space for each subject [29].

### Statistical analysis

Clinical characteristics were compared between CN and CI, and between MCI and AD dementia using the chi-square and Fisher exact tests to compare data distributions, and independent *t* test to compare means of continuous variables. Interobserver agreement for stenosis was determined by calculating Cohen’s kappa correlation coefficient from 125 randomly selected individuals.

The association between a measure of extracranial carotid or intracranial stenosis and AD biomarkers (i.e., global Aβ deposition, AD-CT, and HVa) was investigated focusing on CN and CI separately, with two steps of analysis including exploratory and confirmative steps. Exploratory univariable analyses were performed with independent *t* test to compare the quantitative values of AD biomarkers between the stenosis+ and stenosis− groups. Cohen’s *d* was determined to imply the effect size of the discrepancy between sequences. The AD biomarkers with *p* < 0.05 in exploratory univariable analyses were selected for the next confirmatory multivariable analyses. Confirmative multivariable analyses using general linear model (GLM) were conducted for the selected biomarker adjusting for age, sex, and APOE4 carrier status for the CN group, and for age, sex APOE4 carrier status, and clinical diagnosis (MCI or AD dementia) for the CI group. The Bonferroni correction method was applied to multiple comparisons using *p* < 0.05/no. of confirmatory analyses within each cognitive group. The same analyses with WMH volume as an additional covariate were also conducted to evaluate the mediating effect of WM lesions.

All statistical analyses except calculating Cohen’s *d* were performed using IBM SPSS Statistics 23 (SPSS Inc., Chicago, IL, USA), and *p* < 0.05 (two-sided) was taken to indicate statistical significance unless otherwise specified. Cohen’s *d* was calculated using effsize package in the R 4.0.0.

## Results

### Characteristics of the participants

The demographic and clinical characteristics of the participants are presented in Table 1. Data on the presence of vessel stenosis and AD biomarkers of the participants are also shown in Table 2. Three cases were excluded from the evaluation of extracranial carotid stenosis due to motion artifacts. The characteristics of MCI and AD dementia subgroups in the CI group are also presented in Table S1 and S2 in Additional file 2. The CI group consisted of 129 subjects with MCI and 70 subjects with AD dementia.

**Table 1:** Demographic and clinical characteristics of participants

Variables	CN (*N* = 281)	CI (*N* = 199)	*p* value
Age, years	69.1 ± 8.1	72.9 ± 7.4	< 0.001*
Females	146 (52.0%)	134 (67.3%)	0.001*
Education, years	11.9 ± 4.8	9.7 ± 4.9	< 0.001*
APOE4 carriers	52 (18.5%)	83 (41.7%)	< 0.001*
Global CDR (0/0.5/1)	281/0/0	0/153/46	< 0.001*
CDR-SOB	0.0 ± 0.1	2.7 ± 2.0	< 0.001*
Hypertension	133 (47.3%)	99 (49.7%)	0.602
Diabetes mellitus	46 (16.4%)	34 (17.1%)	0.836
Coronary artery disease	16 (5.7%)	10 (5.0%)	0.470
Hyperlipidemia	96 (34.2%)	71 (35.7%)	0.731
Stroke	0 (0.0%)	0 (0.0%)	NA
Transient ischemic attack	2 (0.7%)	1 (0.5%)	0.774
Vascular risk factor score	17.4 ± 16.11	18.0 ± 16.8	0.679
WMH volume (cm^3^)^a^	5.7 ± 5.3	6.4 ± 5.2	0.202

Data are presented as mean ± SD or *n* (%)

*CN* cognitively normal, *CI* cognitively impaired, *MCI* mild cognitive impairment, *AD* dementia, Alzheimer’s dementia, *CDR-SOB* Clinical Dementia Rating sum of box, *WMH* white matter hyperintensities

^a^Data for 422 individuals were available (256 CN and 166 CI)

*p < 0.05

**Table 2:** Vessel stenosis features and AD biomarkers of participants

Variables	CN (*N* = 281)	CI (*N* = 199)	*p* value
*Large vessel stenosis*			
Presence of any detectable stenosis			
Any extracranial carotid stenosis	23 (8.2%)	22 (11.2%)^a^	0.264
Any intracranial stenosis	77 (27.4%)	71 (35.7%)	0.053
Both extracranial carotid and intracranial stenosis	12 (4.3%)	10 (5.0%)	0.697
Presence of stenosis based on severity threshold			
≥ 50% intracranial stenosis	19 (6.8%)	21 (10.6%)	0.139
Number of stenotic intracranial arteries ≥ 2	44 (15.7%)	38 (19.1%)	0.324
Presence of stenosis according to location			
Anterior circulation stenosis	69 (24.6%)	61 (30.7%)	0.139
Posterior circulation stenosis	21 (7.5%)	21 (10.6%)	0.239
*AD biomarkers*			
Global Aβ deposition (SUVR)	1.184 ± 0.239	1.621 ± 0.156	< 0.001*
Aβ positivity	39 (13.9%)	112 (56.3%)	< 0.001*
Neurodegeneration biomarkers			
AD-CT (mm)	2.866 ± 0.174	2.584 ± 0.286	< 0.001*
HVa (mm^3^)	− 759 ± 838	− 2132 ± 1219	< 0.001*

Data are presented as mean ± SD or *n* (%)

*CN* cognitively normal, *CI* cognitively impaired, *AD-CT* Alzheimer’s disease-signature cortical thickness, *HVa* hippocampal volume adjusted for intracranial volume

^a^Among CI group, data for 196 individuals were available

*p < 0.05

### Reproducibility of a measure of extracranial carotid or intracranial stenosis

The results of the interobserver agreement for a measure of extracranial carotid or intracranial stenosis are shown as Table S3 in Additional file 2. The kappa values were 0.715 for extracranial carotid stenosis, 0.869 for any intracranial stenosis, 0.715 for number of stenotic intracranial arteries ≥ 2, 0.802 for anterior circulation stenosis, and 1 for posterior circulation stenosis. The kappa value for ≥ 50% stenosis was 0.301 due to the very low prevalence of ≥ 50% stenosis despite the high degree of interobserver agreement.

### Association of carotid and intracranial stenosis with AD biomarkers in the CN group

In the exploratory step of the analyses, we found no significant differences in global Aβ deposition between CN subjects with vs. those without any type of stenosis (Table 3). In contrast, with regard to neurodegeneration biomarkers, AD-CT was significantly reduced in CN subjects in the stenosis+ group compared with CN subjects in the stenosis− group for the presence of any extracranial carotid stenosis, presence of any intracranial stenosis, number of stenotic intracranial arteries ≥ 2, anterior circulation stenosis, and posterior circulation stenosis (all *p* < 0.05; Table 3). In addition, HVa was significantly reduced in the stenosis+ CN group compared to the stenosis− CN group for the presence of any intracranial arterial stenosis. Next, for confirmatory analyses in the CN group, we further investigated the associations between AD-CT and each of the abovementioned five measurements of stenosis that showed association in exploratory univariable analyses (*p* < 0.05), as well as the association between HVa and presence of any intracranial stenosis, after controlling for the effects of age, sex, and APOE4 carrier status (Table 4). When Bonferroni-corrected *p* value (*p* < 0.05/6 = 0.008) was applied, these associations were not significant in the confirmatory analyses, although posterior circulation stenosis showed a trend for association with reduced AD-CT (*p* = 0.030).

**Table 3:** Exploratory univariable analyses for the association between extracranial carotid and intracranial arterial stenosis and AD biomarkers in the CN group

**AD biomarker**	**Variables**	**Presence of any detectable stenosis**							
		**Any extracranial carotid stenosis**				**Any intracranial stenosis**			
		**Stenosis−**	**Stenosis+**	***p*** **value**	***d***^a^	**Stenosis−**	**Stenosis+**	***p*** **value**	***d***
Aβ biomarker	Global Aβ	1.185 ± 0.245	1.173 ± 0.160	0.810	0.052	1.182 ± 0.243	1.190 ± 0.230	0.782	− 0.037
Neurodegeneration biomarker	AD-CT	2.877 ± 0.170	2.743 ± 0.173	< 0.001*	0.786	2.890 ± 0.171	2.804 ± 0.165	< 0.001*	0.505
	HVa	− 732 ± 810	− 1067 ± 1076	0.066	0.401	− 686 ± 832	− 953 ± 0827	0.017*	0.322
		**Presence of stenosis based on severity threshold**							
		**≥ 50% intracranial stenosis**^b^				**Number of stenotic intracranial arteries ≥ 2**^c^			
		**Stenosis−**	**Stenosis+**	***p*** **value**	***d***	**Stenosis−**	**Stenosis+**	***p*** **value**	***d***
Aβ biomarker	Global Aβ	1.179 ± 0.234	1.258 ± 0.293	0.165	− 0.331	1.189 ± 0.242	1.157 ± 0.221	0.409	0.136
Neurodegeneration biomarker	AD-CT	2.871 ± 0.175	2.801 ± 0.136	0.091	0.403	2.882 ± 0.169	2.780 ± 0.178	< 0.001*	0.603
	HVa	− 746 ± 838	− 937 ± 837	0.338	0.228	− 726 ± 840	− 938 ± 810	0.123	0.254
		**Presence of stenosis according to location**							
		**Anterior circulation**				**Posterior circulation**			
		**Stenosis−**	**Stenosis+**	***p*** **value**	***d***	**Stenosis−**	**Stenosis+**	***p*** **value**	***d***
Aβ biomarker	Global Aβ	1.187 ± 0.245	1.175 ± 0.220	0.711	0.052	1.185 ± 0.241	1.175 ± 0.212	0.854	0.042
Neurodegeneration biomarker	AD-CT	2.884 ± 0.172	2.811 ± 0.167	0.002*	0.428	2.876 ± 0.170	2.744 ± 0.181	0.001*	0.772
	HVa	− 710 ± 837	− 911 ± 828	0.082	0.242	− 760 ± 836	− 746 ± 875	0.943	− 0.016

Data for continuous variables presented as means ± SD (SUVR for global Aβ, mm for AD-CT, and mm^3^ for HVa)

*CN* cognitively normal, *SUVR* standardized uptake value ratio, *AD-CT* Alzheimer’s disease-signature cortical thickness, *HVa* hippocampal volume adjusted for intracranial volume

^a^*d* indicates Cohen’s delta

^b^“Stenosis−” group of “≥ 50% intracranial stenosis” means individuals with no stenosis or < 50% intracranial stenosis

^c^“Stenosis−” group of “number of stenotic intracranial arteries ≥ 2” means patients with no stenosis or only one stenotic intracranial artery

*p < 0.05

**Table 4:** Confirmatory multivariable analyses for the association between extracranial carotid and intracranial arterial stenosis and neurodegeneration biomarkers in the CN group

AD biomarker	Variables	Type of stenosis	***B***	SE	***p***
Neurodegeneration biomarker	AD-CT	Any extracranial carotid stenosis	− 0.051	0.033	0.121
		Any intracranial stenosis	− 0.033	0.020	0.104
		Number of stenotic intracranial arteries ≥ 2	− 0.040	0.025	0.113
		Anterior circulation	− 0.024	0.021	0.254
		Posterior circulation	− 0.074	0.034	0.030*
	HVa	Any intracranial stenosis	− 30.760	95.491	0.748

Covariates for confirmatory multivariable analyses include age, sex, and APOE4 carrier status. Units for AD biomarker variables are as follows: SUVR for global Aβ, mm for AD-CT, and mm^3^ for HVa. When Bonferroni-corrected *p* value (*p* < 0.05/6 = 0.008) was applied for confirmatory multivariable analyses regarding neurodegeneration biomarkers, these associations were not significant, although posterior circulation stenosis showed a trend for association with reduced AD-CT

*AD* Alzheimer’s disease, *CN* cognitively normal, *SE* standard error, *AD-CT* Alzheimer’s disease-signature cortical thickness, *HVa* hippocampal volume adjusted for intracranial volume

^a^No significant findings in the exploratory univariable analyses step regarding Aβ biomarker

*p < 0.05 (before Bonferroni correction)

### Association of carotid and intracranial stenosis with AD biomarkers in the CI group

**Fig. 1:**
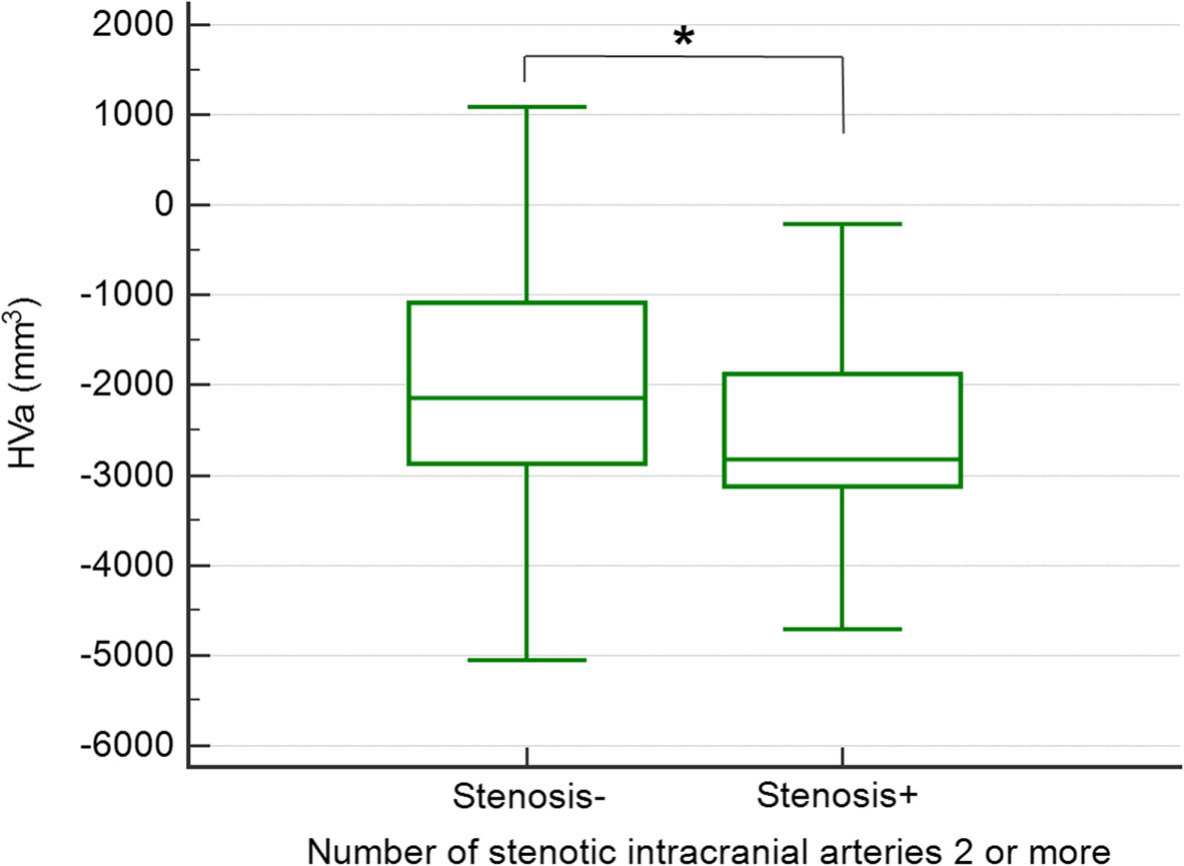
Comparison of HVa between the stenosis− and stenosis+ groups for number of stenotic intracranial arteries ≥ 2 in CI subjects. In the box-and-whisker plot, the central box represents the values from the lower to upper quartile, the middle line represents the median, and the horizontal line extends from the minimum to the maximum value. *Adjusted *p* < 0.05 (after controlling for the effects of age, gender, APOE4, and clinical diagnosis (MCI vs. AD dementia)). HVa, hippocampal volume adjusted for intracranial volume; MCI, mild cognitive impairment; AD, Alzheimer’s disease

**Fig. 2:**
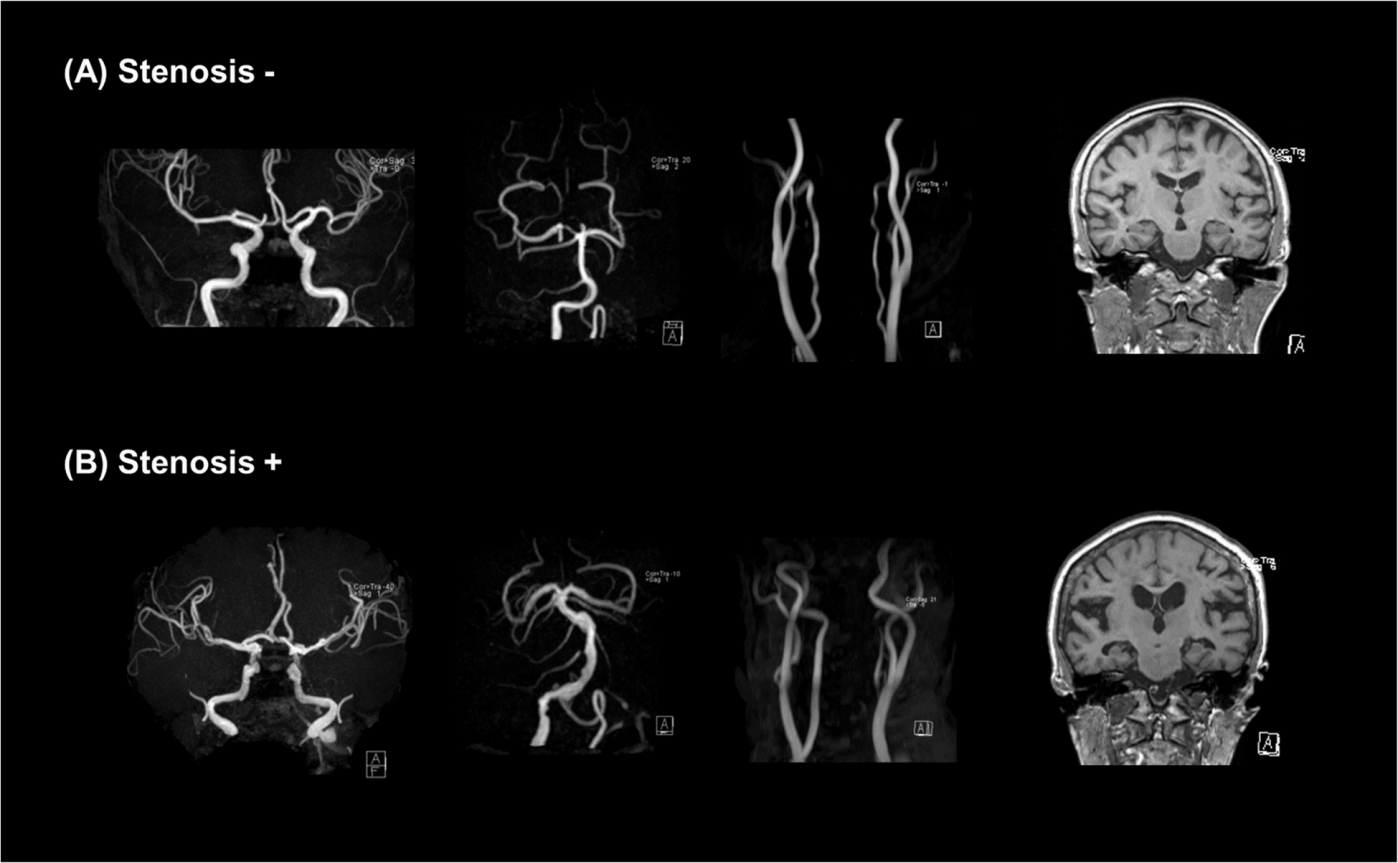
Representative MR angiographic images of intracranial and neck vessels, and coronal sections of T1-weighted images showing medial temporal structures including the bilateral hippocampi of CI individuals in the **a** stenosis− group and **b** stenosis+ group with regard to number of stenotic intracranial arteries ≥ 2. **a** Stenosis−: MR angiography of a 77-year-old woman with MCI with no steno-occlusive lesions in both intracranial and neck vessels. HVa was − 1052 mm^3^, and no significant hippocampal atrophy was observed in coronal sections on T1-weighted MRI. **b** Stenosis+: MR angiography of an 81-year-old woman with MCI with multifocal intracranial arterial stenosis, while no steno-occlusive lesions were found in the extracranial carotid arteries. HVa was − 3862 mm^3^, and coronal sections on T1-weighted MRI indicate bilateral hippocampal atrophy. MR, magnetic resonance; HVa, hippocampal volume adjusted for intracranial volume; MCI, mild cognitive impairment

In the CI group, exploratory univariable analyses found an association between anterior circulation stenosis and lower global Aβ deposition (*p* = 0.049, Table 5). However, it was not significant in the confirmatory step when controlling for age, gender, APOE4 carrier status, and clinical diagnosis (MCI vs. AD dementia) (*B* = − 0.096, SE = 0.072, and *p* = 0.183). In terms of exploratory univariable analyses of neurodegeneration biomarkers, there were no differences in AD-CT between the stenosis+ and stenosis− groups for any type of stenosis in CI subjects (Table 5). However, the presence of any intracranial arterial stenosis and number of stenotic intracranial arteries ≥ 2 were associated with reduced HVa in CI subjects (*p* = 0.047 and *p* = 0.008, respectively; Table 5). These two associations were selected for subsequent confirmatory multivariable analyses after controlling for age, gender, APOE4 carrier status, and clinical diagnosis (Table 6). Bonferroni-corrected *p* < 0.05/2 = 0.025 was used as a statistical threshold. In the confirmatory multivariable analyses, CI subjects with number of stenotic intracranial arteries ≥ 2 had significantly lower HVa than those without when controlling for age, gender, APOE4 carrier status, and clinical diagnosis (*p* = 0.021; Figs. 1 and 2). The results did not change even after additional adjustment for WMH volume (*B* = − 509.45, SE = 205.55, and *p* = 0.014). In order to investigate whether the presence of Aβ deposition (i.e., Aβ positivity) moderates the association between the stenosis and lower HVa, we additionally tested similar GLM model including the stenosis (i.e., number of stenotic intracranial arteries ≥ 2) × Aβ positivity interaction term, as well as the stenosis itself, as an independent variable. In this analysis, we did not find any significant stenosis × Aβ positivity interaction effect. On the other hand, the association between presence of any intracranial stenosis and lower HVa in the CI group did not remain significant in confirmatory analysis (*p* = 0.597; Table 6).

**Table 5:** Exploratory univariable analyses for the association between extracranial carotid and intracranial arterial stenosis and AD biomarkers in the CI group

**AD biomarker**	**Variables**	**Presence of any detectable stenosis**							
		**Any extracranial carotid stenosis**				**Any intracranial stenosis**			
		**Stenosis−**	**Stenosis+**	***p*** **value**	***d***^a^	**Stenosis−**	**Stenosis+**	***p*** **value**	***d***
Aβ biomarker	Global Aβ	1.627 ± 0.530	1.609 ± 0.412	0.857	0.334	1.648 ± 0.539	1.573 ± 0.472	0.304	0.147
Neurodegeneration biomarker	AD-CT	2.600 ± 0.284	2.518 ± 0.260	0.205	0.288	2.596 ± 0.311	2.561 ± 0.234	0.376	0.121
	HVa	− 2064 ± 1225	− 2396 ± 947	0.146	0.277	− 2004 ± 1262	− 2362 ± 1109	0.047*	0.296
		**Presence of stenosis based on severity threshold**							
		**≥ 50% intracranial stenosis**^b^				**Number of stenotic intracranial arteries ≥ 2**^c^			
		**Stenosis−**	**Stenosis+**	***p*** **value**	***d***	**Stenosis−**	**Stenosis+**	***p*** **value**	***d***
Aβ biomarker	Global Aβ	1.646 ± 0.522	1.415 ± 0.411	0.052	0.451	1.640 ± 0.536	1.544 ± 0.414	0.234	0.185
Neurodegeneration biomarker	AD-CT	2.584 ± 0.290	2.579 ± 0.254	0.940	0.017	2.598 ± 0.292	2.521 ± 0.252	0.136	0.270
	HVa	− 2087 ± 1202	− 2515 ± 1327	0.128	0.352	− 2021 ± 1227	− 2602 ± 1080	0.008*	0.484
		**Presence of stenosis according to location**							
		**Anterior circulation**				**Posterior circulation**			
		**Stenosis−**	**Stenosis+**	***p*** **value**	***d***	**Stenosis−**	**Stenosis+**	***p*** **value**	***d***
Aβ biomarker	Global Aβ	1.667 ± 0.535	1.519 ± 0.456	0.049*	0.288	1.623 ± 0.519	1.605 ± 0.498	0.878	0.035
Neurodegeneration biomarker	AD-CT	2.585 ± 0.309	2.580 ± 0.224	0.902	0.017	2.589 ± 0.289	2.540 ± 0.257	0.459	0.171
	HVa	− 2047 ± 1244	− 2323 ± 1147	0.142	0.227	− 2100 ± 1217	− 2400 ± 1236	0.287	0.246

Data for continuous variables presented as means ± SD (SUVR for global Aβ, mm for AD-CT, and mm^3^ for HVa)

*CI* cognitively impaired, *SUVR* standardized uptake value ratio, *AD-CT* Alzheimer’s disease-signature cortical thickness, *HVa* hippocampal volume adjusted for intracranial volume

^a^*d* indicates Cohen’s delta

^b^“Stenosis−” group of “≥50% intracranial stenosis” means individuals with no stenosis or < 50% intracranial stenosis

^c^“Stenosis−” group of “number of stenotic intracranial arteries ≥ 2” means patients with no stenosis or only one stenotic intracranial artery

*p < 0.05

**Table 6:** Confirmatory multivariable analyses for the association between extracranial carotid and intracranial arterial stenosis and HVa in the CI group

AD biomarker	Variables	Type of stenosis	***B***	SE	***p***
Neurodegeneration biomarker	HVa	Any intracranial stenosis	− 84.119	158.679	0.597
		Number of stenotic intracranial arteries ≥ 2	− 428.816	184.921	0.021*^†^

Covariates for confirmatory multivariable analyses include age, sex, APOE4 carrier status, and clinical diagnosis (mild cognitive impairment or AD dementia). Units for AD biomarker variables are as follows: SUVR for global Aβ, mm for AD-CT, and mm^3^ for HVa. When Bonferroni-corrected *p* value was applied for confirmatory multivariable analyses regarding neurodegeneration biomarkers, the association between presence of number of stenotic intracranial arteries ≥ 2 and lower HVa in the CI group remained significant

*AD* Alzheimer’s disease, *CI* cognitively impaired, *SE* standard error, *SUVR* standardized uptake value ratio, *HVa* hippocampal volume adjusted for intracranial volume

^a^No significant findings in the exploratory univariable analyses step regarding Aβ biomarker and AD-CT

*p < 0.05 (before Bonferroni correction)

^†^*p* < 0.025 (Bonferroni-corrected *p* < 0.05/2 = 0.025 was used as a statistical threshold)

## Discussion

This study was performed to investigate the associations of both extracranial carotid and intracranial artery stenosis with in vivo AD pathologies, i.e., global Aβ burden and neurodegeneration in a large number of older adults, focusing on the CN and CI groups separately. Global Aβ burden was not related to any vessel stenosis in either group. With regard to neurodegeneration, the CN group did not show any significant associations between carotid and intracranial artery stenosis and AD-CT or HVa. In contrast, in the CI group, number of stenotic intracranial arteries ≥ 2 was significantly associated with lower HVa even after adjusting for age, gender, APOE4 carrier status, and diagnosis (i.e., MCI vs. AD dementia).

A number of previous *postmortem* brain studies investigated the association between cerebral atherosclerosis and Aβ burden, but the results were controversial [1–8]. While a number of previous autopsy studies reported a significant association between intracranial atherosclerosis and neuritic plaques in AD [1–4], several others found no such associations [5–8]. Recently, a community-based study in adults without dementia reported that intracranial atherosclerotic plaque or stenosis was not associated with Aβ deposition in the brain [14]. Our results in the CN and CI groups are in agreement with the study [14] regarding the relationship between carotid and intracranial stenosis and global Aβ burden. As both cerebral atherosclerosis and Aβ deposition are common in the elderly, the possibility of coincidence cannot be excluded in *postmortem* studies with positive association between the two.

In contrast to global Aβ burden, intracranial artery stenosis was associated with neurodegeneration biomarkers in the CI group. In particular, number of stenotic intracranial arteries ≥ 2 was significantly associated with lower hippocampal volume in CI subjects in confirmatory multivariable analysis. Some previous studies identified intracranial atherosclerosis as an independent risk factor for cerebral atrophy [5, 30]. In these studies, however, intracranial atherosclerosis was assessed based only on cavernous ICA calcification on computed tomography [30] or pathological examination of the circle of Willis [31]. In contrast, we evaluated stenosis of all cerebral arteries. With this approach and strict control for multiple testing errors, we confirmed the association between intracranial artery stenosis and HVa in the CI group. The association remained significant even after controlling for WMH volume, suggesting that intracranial artery stenosis affects hippocampal atrophy independently of changes in the WM. Hippocampal atrophy is a validated neurodegeneration biomarker of AD and is closely correlated with early cognitive decline in AD [32]. Therefore, the association of intracranial stenosis with decreased HVa, together with no association with Aβ burden, in the CI group indicates that intracranial stenosis contributes to the development of CI via Aβ-independent neurodegeneration of the hippocampus.

In the CN group, while exploratory univariable analyses showed relations between various types of stenosis and AD-CT or HVa in CN, such relations were not confirmed by multivariable analyses with a strict statistical threshold. Nevertheless, AD-CT tended to be lower in subjects with posterior circulation stenosis than those without (*p* = 0.03), although the difference was not statistically significant after Bonferroni correction. In a previous *postmortem* study which reported the association between intracranial artery atherosclerosis and AD dementia, the posterior cerebral artery (PCA), one of the posterior circulation, showed the most severe atherosclerosis among the circle of Willis arteries in AD dementia patients [7]. Therefore, the potential association between posterior circulation stenosis and AD-CT deserves attention. Actually, the AD-signature regions, where we measured cortical thickness, include the entorhinal, inferior temporal, and fusiform gyri that receive blood supply from the PCA.

We found no significant associations between extracranial carotid stenosis and AD biomarkers regardless of the presence of cognitive impairment. Some previous in vivo studies with very small sample sizes investigated the associations between severe carotid stenosis or occlusion and Aβ deposition and yielded contradictory findings. While one study using [^18^F] AV-45 PET indicated that Aβ deposition increased in dementia patients with unilateral carotid artery stenosis, and its distribution was lateralized to the side of stenosis [12], a more recent study using [^18^F] flutemetamol PET indicated that cerebral hypoperfusion caused by unilateral occlusion of the ICA did not induce brain accumulation of Aβ [13]. In terms of neurodegeneration, our results were inconsistent with several previous studies that showed increased carotid intima–media thickness and carotid stenosis were associated with decreased total brain volume [8, 33]. This discrepancy may have been due to the severity of stenosis. Only a very small number of subjects with ≥ 50% stenosis in the extracranial carotid artery were included in the present study (1 of 281 subjects in the CN group and 1 of 196 subjects in the CI group), while many previous studies indicated associations of the degree of severity [3, 33] or severe carotid atherosclerosis [34, 35] with brain atrophy.

To our knowledge, this is the first study to investigate the association between MR angiography-measured vessel stenosis in the extracranial carotid and intracranial arteries and in vivo AD pathologies, including both Aβ deposition and neurodegeneration, in a large sample of older adults, focusing separately on the CN and CI groups. However, there were some limitations in our study. First, due to its cross-sectional design, we cannot make conclusions regarding the cause and effect relationship between carotid and intracranial artery stenosis and in vivo AD pathologies. Second, although we included a relatively large number of subjects, the frequencies of stenosis, particularly extracranial carotid stenosis, number of stenotic intracranial arteries ≥ 2, and posterior circulation stenosis, were relatively low in both the CN and CI groups, which may have reduced statistical power and made it difficult to identify significant associations after multiple comparison correction. Carotid and intracranial artery stenosis has not been a common finding in community-dwelling subjects [36, 37], with reported prevalence rates of asymptomatic intracranial stenosis ranging from 5.9 to 24.5% [37]. In addition, the prevalence of cervical carotid artery stenosis varies significantly with ethnicity, and it was reported to be particularly uncommon in a Korean population-based screening cohort [36]. Therefore, our study population seemed to reflect the prevalence of asymptomatic carotid and intracranial stenosis in the general Asian population. Finally, given that about 55% of MCI individuals and 23% of clinically defined AD dementia patients among the subjects were Aβ negative, brain pathological conditions other than AD and vascular lesions, such as primary age-related taupathy, hippocampal sclerosis, and argyrophilic grain disease, might contribute to neurodegenerative changes. Future studies including the examination of the conditions are needed.

## Conclusions

In conclusion, our findings suggested that neither carotid nor intracranial artery stenosis is associated with brain Aβ burden, while intracranial artery stenosis is related to amyloid-independent neurodegeneration, particularly hippocampal atrophy.

## Supplementary information

**Additional file 1.** Supplementary methods (Method S1 and S2).

**Additional file 2.** Supplementary tables (Table S1, S2, and S3).

## Data Availability

The datasets generated and analyzed during the present study are not publicly available, owing to ethics considerations and privacy restriction. Data may be available from the corresponding author once approval from the Institutional Review Board of the Seoul National University Hospital, South Korea, has been sought.

## Abbreviations

3D: Three-dimensional
AAL: Automatic anatomic labeling
Aβ: Beta-amyloid
AD: Alzheimer’s disease
AD-CT: AD-signature cortical thickness
ANVOCA: Analysis of covariance
APOE4: Apolipoprotein E ε4 allele
CDR: Clinical Dementia Rating
CERAD-K: Consortium to Establish a Registry for Alzheimer’s Disease
CI: Cognitive impairment
CN: Cognitively normal
DSM-IV-TR: Diagnostic and Statistical Manual of Mental Disorders 4th Edition
GM: Gray matter
HV: Hippocampal volume
HVa: Hippocampal volume adjusted for intracranial volume
IBMSPM: Individual Brain Atlases using Statistical Parametric Mapping
ICA: Internal carotid artery
ICV: Intracranial volume
KBASE: Korean Brain Aging Study for Early Diagnosis and Prediction of Alzheimer’s Disease
MCI: Mild cognitive impairment
MNI: Montreal Neurological Institute
MR: Magnetic resonance
NASCET: North American Symptomatic Carotid Endarterectomy Trial
NIA-AA: National Institute on Aging-Alzheimer’s Association
PET: Positron emission tomography
PiB: Pittsburgh compound B
ROIs: Regions of interest
SE: Standard error
SPM: Statistical Parametric Mapping
SUVR: Standardized uptake value ratio
TOF: Time-of-flight
WM: White matter
WMH: White matter hyperintensities

## References

[1] Hofman A, Ott A, Breteler MM, Bots ML, Slooter AJ, van Harskamp F (1997). Atherosclerosis, apolipoprotein E, and prevalence of dementia and Alzheimer’s disease in the Rotterdam Study. Lancet.

[2] Silvestrini M, Gobbi B, Pasqualetti P, Bartolini M, Baruffaldi R, Lanciotti C (2009). Carotid atherosclerosis and cognitive decline in patients with Alzheimer’s disease. Neurobiol Aging.

[3] Roher AE, Garami Z, Tyas SL, Maarouf CL, Kokjohn TA, Belohlavek M (2011). Transcranial Doppler ultrasound blood flow velocity and pulsatility index as systemic indicators for Alzheimer’s disease. Alzheimers Dement.

[4] Arvanitakis Z, Capuano AW, Leurgans SE, Bennett DA, Schneider JA (2016). Relation of cerebral vessel disease to Alzheimer’s disease dementia and cognitive function in elderly people: a cross-sectional study. Lancet Neurol.

[5] Honig LS, Kukull W, Mayeux R (2005). Atherosclerosis and AD: analysis of data from the US National Alzheimer’s Coordinating Center. Neurology.

[6] Beach TG, Wilson JR, Sue LI, Newell A, Poston M, Cisneros R (2007). Circle of Willis atherosclerosis: association with Alzheimer’s disease, neuritic plaques and neurofibrillary tangles. Acta Neuropathol.

[7] Roher AE, Tyas SL, Maarouf CL, Daugs ID, Kokjohn TA, Emmerling MR (2011). Intracranial atherosclerosis as a contributing factor to Alzheimer’s disease dementia. Alzheimers Dement.

[8] Yarchoan M, Xie SX, Kling MA, Toledo JB, Wolk DA, Lee EB (2012). Cerebrovascular atherosclerosis correlates with Alzheimer pathology in neurodegenerative dementias. Brain.

[9] Kosunen O, Talasniemi S, Lehtovirta M, Heinonen O, Helisalmi S, Mannermaa A (1995). Relation of coronary atherosclerosis and apolipoprotein E genotypes in Alzheimer patients. Stroke.

[10] Itoh Y, Yamada M, Sodeyama N, Suematsu N, Matsushita M, Otomo E (1999). Atherosclerosis is not implicated in association of APOE ε4 with AD. Neurology.

[11] Dolan H, Crain B, Troncoso J, Resnick SM, Zonderman AB, Obrien RJ (2010). Atherosclerosis, dementia, and Alzheimer disease in the Baltimore Longitudinal Study of Aging cohort. Ann Neurol.

[12] Huang K-L, Lin K-J, Ho M-Y, Chang Y-J, Chang C-H, Wey S-P (2012). Amyloid deposition after cerebral hypoperfusion: evidenced on [18 F] AV-45 positron emission tomography. J Neurol Sci.

[13] Hansson O, Palmqvist S, Ljung H, Cronberg T, van Westen D, Smith R. Cerebral hypoperfusion is not associated with an increase in amyloid β pathology in middle-aged or elderly people. Alzheimers Dement. 2018;14(1):54–61. 10.1016/j.jalz.2017.06.2265 PMC5766833 28719802

[14] Gottesman RF, Mosley TH, Knopman DS, Hao Q, Wong D, Wagenknecht LE, et al. Association of intracranial atherosclerotic disease with brain beta-amyloid deposition: secondary analysis of the ARIC study. JAMA Neurol. 2019;77(3):350–7. 10.1001/jamaneurol.2019.4339 PMC6990749 31860001

[15] Rowe CC, Ng S, Ackermann U, Gong SJ, Pike K, Savage G (2007). Imaging β-amyloid burden in aging and dementia. Neurology.

[16] Byun MS, Yi D, Lee JH, Choe YM, Sohn BK, Lee J-Y (2017). Korean brain aging study for the early diagnosis and prediction of Alzheimer’s disease: methodology and baseline sample characteristics. Psychiatry Investig.

[17] Albert MS, DeKosky ST, Dickson D, Dubois B, Feldman HH, Fox NC (2011). The diagnosis of mild cognitive impairment due to Alzheimer’s disease: recommendations from the National Institute on Aging-Alzheimer’s Association workgroups on diagnostic guidelines for Alzheimer's disease. Alzheimers Dement.

[18] McKhann GM, Knopman DS, Chertkow H, Hyman BT, Jack CR, Kawas CH (2011). The diagnosis of dementia due to Alzheimer’s disease: recommendations from the National Institute on Aging-Alzheimer’s Association workgroups on diagnostic guidelines for Alzheimer’s disease. Alzheimers Dement.

[19] Fox AJ (1993). How to measure carotid stenosis. Radiology.

[20] Samuels OB, Joseph GJ, Lynn MJ, Smith HA, Chimowitz MI (2000). A standardized method for measuring intracranial arterial stenosis. Am J Neuroradiol.

[21] Tzourio-Mazoyer N, Landeau B, Papathanassiou D, Crivello F, Etard O, Delcroix N (2002). Automated anatomical labeling of activations in SPM using a macroscopic anatomical parcellation of the MNI MRI single-subject brain. Neuroimage.

[22] Lopresti BJ, Klunk WE, Mathis CA, Hoge JA, Ziolko SK, Lu X (2005). Simplified quantification of Pittsburgh Compound B amyloid imaging PET studies: a comparative analysis. J Nucl Med.

[23] Reiman EM, Chen K, Liu X, Bandy D, Yu M, Lee W (2009). Fibrillar amyloid-beta burden in cognitively normal people at 3 levels of genetic risk for Alzheimer’s disease. Proc Natl Acad Sci U S A.

[24] Jack CR, Wiste HJ, Weigand SD, Knopman DS, Mielke MM, Vemuri P (2015). Different definitions of neurodegeneration produce similar amyloid/neurodegeneration biomarker group findings. Brain.

[25] Choe YM, Sohn BK, Choi HJ, Byun MS, Seo EH, Han JY (2014). Association of homocysteine with hippocampal volume independent of cerebral amyloid and vascular burden. Neurobiol Aging.

[26] Desikan RS, Segonne F, Fischl B, Quinn BT, Dickerson BC, Blacker D (2006). An automated labeling system for subdividing the human cerebral cortex on MRI scans into gyral based regions of interest. Neuroimage.

[27] Lee JH, Byun MS, Yi D, Choe YM, Choi HJ, Baek H (2017). Sex-specific association of sex hormones and gonadotropins, with brain amyloid and hippocampal neurodegeneration. Neurobiol Aging.

[28] Tsai J-Z, Peng S-J, Chen Y-W, Wang K-W, Li C-H, Wang J-Y (2014). Automated segmentation and quantification of white matter hyperintensities in acute ischemic stroke patients with cerebral infarction. PLoS One.

[29] Kochunov P, Lancaster JL, Thompson P, Woods R, Mazziotta J, Hardies J (2001). Regional spatial normalization: toward an optimal target. J Comput Assist Tomogr.

[30] Erbay S, Han R, Aftab M, Zou KH, Polak J, Bhadelia RA (2008). Is intracranial atherosclerosis an independent risk factor for cerebral atrophy? A retrospective evaluation. BMC Neurol.

[31] A Crystal H, A Schneider J, A Bennett D, Leurgans S, R Levine S. Associations of cerebrovascular and Alzheimer’s disease pathology with brain atrophy. Curr Alzheimer Res 2014, 11(4):309–316. 10.2174/1567205011666140302194358 PMC4401460 24597507

[32] Sperling RA, Aisen PS, Beckett LA, Bennett DA, Craft S, Fagan AM (2011). Toward defining the preclinical stages of Alzheimer’s disease: recommendations from the National Institute on Aging-Alzheimer’s Association workgroups on diagnostic guidelines for Alzheimer’s disease. Alzheimers Dement.

[33] Kin T, Yamano S, Sakurai R, Kajitani M, Okahashi Y, Nishiura N (2007). Carotid atherosclerosis is associated with brain atrophy in Japanese elders. Gerontology.

[34] Bos D, Vernooij MW, Elias-Smale SE, Verhaaren BF, Vrooman HA, Hofman A (2012). Atherosclerotic calcification relates to cognitive function and to brain changes on magnetic resonance imaging. Alzheimers Dement.

[35] Muller M, van der Graaf Y, Algra A, Hendrikse J, Mali WP, Geerlings MI (2011). Carotid atherosclerosis and progression of brain atrophy: the SMART-MR study. Ann Neurol.

[36] Woo SY, Joh JH, Han S-A, Park H-C. Prevalence and risk factors for atherosclerotic carotid stenosis and plaque: a population-based screening study. Medicine (Baltimore). 2017;96(4):e5999. 10.1097/MD.0000000000005999 PMC5287981 28121957

[37] Arenillas JF (2011). Intracranial atherosclerosis: current concepts. Stroke.

