# Supplementary material for: Association of carotid and intracranial stenosis with Alzheimer’s disease biomarkers
Source: Alzheimers Res Ther. 2020 Sep 10;12:106. doi: 10.1186/s13195-020-00675-6 (PMC7488394; doi:10.1186/s13195-020-00675-6)
Supplement: Supplementary file 1 — Additional file 1. Supplementary methods (Method S1 and S2). [file 13195_2020_675_MOESM1_ESM.docx]

**Additional file 1.**

**Methods S1. Acquisition parameters of magnetic resonance imaging (MRI) and magnetic resonance angiography (MRA)**

3D T1-weighted images and fluid attenuated inversion recovery (FLAIR) images were

acquired in the sagittal plane. Acquisition parameters of T1 weighted MRI were as follows:

repetition time (TR), 1,670ms; echo time (TE), 1 89ms; field of view (FOV), 250mm; matrix, 256 × 256; slice thickness, 1.0mm. The parameters for acquiring FLAIR images were as follows: TR, 5,000ms; TE, 173ms; echo spacing, 3.46ms; FOV, 250 mm; matrix size, 256 × 256; slice thickness, 1.0 mm.

Acquisition parameters for 3D Time-of-Flight (TOF) MRA for the major intracranial vessels were as follows: field of view (FOV), 220 × 178 mm^2^ (frequency × phase); acquisition matrix, 512 × 208 (frequency × phase); pixel size, 0.43 × 0.86 mm^2^ (frequency × phase); slice thickness, 0.6 mm. In the case of the neck vessels, acquisition parameters were as follows: FOV, 220 × 176mm^2^ (frequency × phase); acquisition matrix, 384 × 200 (frequency × phase); pixel size, 0.57 × 0.88 mm^2^ (frequency × phase); slice thickness, 1. 6 mm.

**Methods S2. Modification of an optimal threshold used for automated white matter hyperintensity (WMH) volume calculation**

As the authors of the methodology study suggested (1), several thresholds (*i.e*., 60, 65, 70, 75) were used to test the optimal threshold that is more suitable for the current data. Post-processing data using the abovementioned threshold options were reviewed by imaging experts and it was deemed that the optimal threshold for the current data was 70 as opposed to 65 from the original study; in other words, using the threshold of 70 captured the voxels with WMH better than 65 without including non-WMH voxels. This difference might be stem from the differences among MRI scanner and images parameters of FLAIR images between our data and the reference (1); All MRI data in the reference (29) were obtained using a Signa HDxt 1.5T (GE healthcare Milwaukee, WI) scanner, while we used a 3.0T Biograph mMR PET-MR scanner. Tsai et al. (1) did imply that using different scanners may require adjusting the thresholds.
