# Supplementary material for: Association of carotid and intracranial stenosis with Alzheimer’s disease biomarkers
Source: Alzheimers Res Ther. 2020 Sep 10;12:106. doi: 10.1186/s13195-020-00675-6 (PMC7488394; doi:10.1186/s13195-020-00675-6)
Supplement: Supplementary file 2 — Additional file 2. Supplementary tables (Table S1, S2, and S3). [file 13195_2020_675_MOESM2_ESM.docx]

**Additional file 2. Supplementary tables**

**Table S1. Demographic and clinical characteristics of MCI and AD dementia participants**

| Variables | MCI  (N=129) | AD dementia  (N = 70) | P value |
| --- | --- | --- | --- |
| Age, y | 73.4 ± 7.0 | 72.0 ± 8.0 | 0.209 |
| Females | 85 (65.9%) | 49 (70.0%) | 0.555 |
| Education, y | 10.0 ± 4.5 | 9.2 ± 5.5 | 0.315 |
| APOE4 carriers | 42 (32.6%) | 41 (58.6%) | < 0.001^*^ |
| Global CDR (0.5/1) | 129 (100%)/0 (0%) | 24 (34.3%)/ 46 (65.7%) | < 0.001^*^ |
| CDR-SOB | 1.5 ± 0.6 | 5.0 ± 1.5 | < 0.001^*^ |
| Hypertension | 70 (54.3%) | 29 (41.4 %) | 0.084 |
| Diabetes Mellitus | 22 (17.1%) | 12 (17.1 %) | 0.987 |
| Coronary artery disease | 5 (3.9 %) | 5 (7.1%) | 0.326 |
| Hyperlipidemia | 49 (38.0%) | 22 (31.4 %) | 0.357 |
| Stroke | 0 (0%) | 0 (0%) | NA |
| Transient ischemic attack | 1 (0.8%) | 0 (0%) | 1 |
| Vascular risk factor score | 19.0 ± 16.8 | 16.2 ± 16.8 | 0.262 |
| WMH volume (cm^3^) ^a^ | 6.7 ± 5.5 | 5.7 ± 4.5 | 0.261 |

Note. Data are presented as mean ± SD or n (%).

^a^Data for 110 MCI and 56 AD dementia were available.

*p < 0.05

MCI, mild cognitive impairment; AD dementia, Alzheimer’s dementia; CDR-SOB, Clinical Dementia Rating sum of box; WMH, white matter hyperintensity

**Table S2. Vessel stenosis features and AD biomarkers of MCI and AD dementia participants**

| Variables | MCI  (N= 129) | AD dementia  (N =70) | *P*- value |
| --- | --- | --- | --- |
| *Large vessel stenosis* |  |  |  |
| Overall presence of detectable stenosis |  |  |  |
| Any extracranial carotid stenosis ^a^ | 13 (10.1%) | 9 (12.9%) | 0.552 |
| Any intracranial stenosis | 46 (35.7) | 25 (35.7%) | 0.994 |
| Both extracranial carotid and intracranial stenosis | 5 (3.9%) | 5 (7.1%) | 0.314 |
| Severity |  |  |  |
| ≥ 50 % intracranial stenosis^b^ | 16 (12.4%) | 5 (7.1%) | 0.249 |
| Number of stenotic intracranial arteries ≥ 2 | 24 (18.6%) | 14 (20.0%) | 0.811 |
| Location |  |  |  |
| Anterior circulation stenosis | 42 (32.6%) | 19 (27.1%) | 0.429 |
| Posterior circulation stenosis | 12 (9.3%) | 9 (12.9%) | 0.436 |
| *AD biomarkers* |  |  |  |
| Global Aβ deposition (SUVR) | 1.492 ± 0.461 | 1.890 ± 0.529 | < 0.001^*^ |
| Aβ positivity | 58 (45.0%) | 54 (77.1%) | < 0.001^*^ |
| Neurodegeneration biomarkers |  |  |  |
| AD-CT(mm) | 2.678 ± 0.253 | 2.409 ± 0.261 | < 0.001^*^ |
| HVa (mm^3^) | -1773 ± 1181 | -2792 ± 998 | < 0.001^*^ |

Note. Data are presented as mean ± SD or n (%).

^a^Data for 127 MCI and 69 AD dementia individuals were available.

*p < 0.05

MCI, mild cognitive impairment; AD dementia, Alzheimer’s dementia; AD-CT, Alzheimer’s disease signature cortical thickness; HVa, Hippocampal volume adjusted for intracranial volume

**Table S3. Interobserver agreement of a measure of extracranial carotid or intracranial stenosis**

| **Any extracranial carotid stenosis** | Observer 1 | | | | **Any intracranial stenosis** | Observer 1 | | | |
| --- | --- | --- | --- | --- | --- | --- | --- | --- | --- |
|  | Stenosis- | Stenosis+ | Total | Kappa |  | Stenosis- | Stenosis+ | Total | Kappa |
| Observer 2 |  |  |  | 0.715 | Observer 2 |  |  |  | 0.869 |
| Stenosis- | 113 | 3 | 116 |  | Stenosis- | 83 | 1 | 84 |  |
| Stenosis+ | 2 | 7 | 9 |  | Stenosis+ | 6 | 35 | 41 |  |
| Total | 115 | 10 | 125 |  | Total | 89 | 36 | 125 |  |
| **≥ 50 % intracranial stenosis** | Observer 1 | | | | **Number of** **stenotic intracranial arteries ≥ 2** | Observer 1 | | | |
|  | Stenosis- | Stenosis+ | Total | Kappa |  | Stenosis- | Stenosis+ | Total | Kappa |
| Observer 2 |  |  |  | 0.301 | Observer 2 |  |  |  | 0.715 |
| Stenosis- | 115 | 3 | 118 |  | Stenosis- | 102 | 7 | 109 |  |
| Stenosis+ | 5 | 2 | 7 |  | Stenosis+ | 2 | 14 | 16 |  |
| Total | 120 | 5 | 125 |  | Total | 104 | 21 | 125 |  |
| **Anterior circulation** | Observer 1 | | | | **Posterior circulation** | Observer 1 | | | |
|  | Stenosis- | Stenosis+ | Total | Kappa |  | Stenosis- | Stenosis+ | Total | Kappa |
| Observer 2 |  |  |  | 0.802 | Observer 2 |  |  |  | 1 |
| Stenosis- | 85 | 3 | 88 |  | Stenosis- | 117 | 0 | 117 |  |
| Stenosis+ | 7 | 30 | 37 |  | Stenosis+ | 0 | 8 | 8 |  |
| Total | 92 | 33 | 125 |  | Total | 117 | 8 | 125 |  |
